# Aberrant neural processing of event boundaries in persons with Parkinson’s disease

**DOI:** 10.1038/s41598-023-36063-x

**Published:** 2023-05-31

**Authors:** Michelle Wyrobnik, Elke van der Meer, Fabian Klostermann

**Affiliations:** 1 Department of Neurology, Motor and Cognition Group, Charité – Universitätsmedizin Berlin, corporate member of Freie Universität Berlin and Humboldt-Universität zu Berlin, Campus Benjamin Franklin (CBF), Hindenburgdamm 30, 12203 Berlin, Germany; 2 Berlin School of Mind and Brain, Humboldt-Universität zu Berlin, Luisenstraße 56, 10117 Berlin, Germany; 3 Institute of Psychology, Humboldt-Universität zu Berlin, Rudower Chaussee 18, 12489 Berlin, Germany

**Keywords:** Perception, Parkinson's disease

## Abstract

The perception of everyday events implies the segmentation into discrete sub-events (i.e. event segmentation). This process is relevant for the prediction of upcoming events and for the recall of recent activities. It is thought to involve dopaminergic networks which are strongly compromised in Parkinson’s disease (PD). Indeed, deficits of event segmentation have been previously shown in PD, but underlying neuronal mechanisms remain unknown. We therefore investigated 22 persons with PD and 22 age-matched healthy controls, who performed an event segmentation task with simultaneous electroencephalography (EEG). Both groups had to indicate by button press the beginning of sub-events within three movies showing persons performing everyday activities. The segmentation performance of persons with PD deviated significantly from that of controls. Neurophysiologically, persons with PD expressed reduced theta (4–7 Hz) activity around identified event boundaries compared to healthy controls. Together, these results point to disturbed event processing in PD. According to functions attributed to EEG activities in particular frequency ranges, the PD-related theta reduction could reflect impaired matching of perceptual input with stored event representations and decreased updating processes of event information in working memory and, thus, event boundary identification.

## Introduction

Parkinson’s disease (PD) is a prevalent neurological movement disorder, pathologically characterized by the degeneration of dopaminergic cells, particularly in the pars compacta of the substantia nigra (SN). The resulting dopamine deficiency in nigrostriatal networks leads to cardinal PD motor symptoms such as tremor, rigidity, and bradykinesia^1^. Concerning coordinative aspects of motor behavior, persons with PD have particular difficulties with the initiation and sequencing of movements. Interestingly, similar, but less overt problems appear to prevail on perceptual levels, for example, regarding the segmentation of the continuous stream of sensory input^2–4^. Possibly, this contributes to problems which persons with PD often experience in everyday routines, since event segmentation is assumed to enable predictions about upcoming events and, thus, flexible adaptations to possible changes in the environment^5^. According to the event segmentation theory (EST), it is assumed that during event segmentation the sensory input perceived from one’s surroundings together with prior knowledge of everyday events (i.e. event knowledge) facilitates an event model, that is, a mental representation of current event characteristics. As soon as new information is presented in the ongoing stream of events and needs to be integrated, for instance, when perceiving an event boundary or when predictions of the mental model are not fulfilled (i.e. a prediction error occurs), the momentarily given model needs to be adjusted through updating processes^6^. Moreover, event segmentation is considered as a key process for organizing continuously perceived input as discrete events in long-term memory, playing a fundamental role for structuring information and keeping it available in everyday life^7,8^.

In the context of event perception, persons with PD show difficulties in processing event sequences and associated updating processes possibly due to impaired fronto-striatal network functions. For instance, compared to controls persons with PD performed worse when they had to order events chronologically and committed more sequencing errors during the generation of sub-events belonging to a superordinate event^9–11^. In addition, in a few behavioral studies PD-related knowledge deficits, that is, declined long-term memory representations of events were discussed. However, other investigators suggested unimpaired event knowledge processing, for example, due to a presumed relative preservation of associated parietal-temporal networks^9,12,13^. Evidently, *knowing* when one sub-event ends and the next one is about to begin (i.e. event knowledge), and retrieving this information is important for event segmentation, possibly leading to according deficits in PD^14,15^. Indeed, one study showed that persons with PD identified event boundaries at different points in time regarding highly familiar everyday activities compared to controls [^4^, but see also ^16^]. However, it remained unclear which mechanisms contributed to this PD-related behavior, possibly relying on disturbed updating or retrieval functions of event representations. In this regard, investigating segmentation-related brain activities could help to delineate altered processing underlying corresponding abnormalities. Accordingly, we analyzed neurophysiological signaling related to the recognition of event boundaries in persons with PD compared to healthy controls.

Interestingly, imaging studies examining neurophysiological correlates of event segmentation in healthy subjects showed widespread activations around the perception of event boundaries, including the angular gyrus, frontal, posterior medial and parahippocampal cortex, as well as hippocampal activity shortly after the perception of event boundaries. Generally, stable brain patterns in these regions were found within sub-events, which were disrupted by transient changes in brain activity induced by event boundaries^8,17–19^. Further, in a recent neurophysiological study, scalp electroencephalographic (EEG) signals were related to event boundary perception in healthy subjects^20^. Specifically, it was shown that the EEG signal was inter-individually similar while watching the same event sequences, whereby the revealed pattern was more stable (i.e. per time-point spatially more similar) within sub-events than across sub-events. Together, these findings suggest that correlates of event boundary processing can be detected as systematic changes of neuronal activities.

Interestingly, brain signals, as reflected by power changes in particular EEG frequency bands, have been associated to mechanisms important for event segmentation. For example, increases of so-called theta activity (4 to 7 Hz oscillatory EEG signals) were shown to be associated with the encoding and retrieval of episodic memory, integration of new information in working memory, and cognitive control^21–23^. In the context of event segmentation, altered theta activity in the PD group could therefore indicate impaired integration and updating processes for event models in working memory. Changes in the alpha band (8 to 12 Hz) power were rather found to be associated with attentional and semantic memory processes as well as with the inhibition of task irrelevant activities^21,24,25^, that is, such alterations could point to problems in the organization and retrieval of event knowledge. Further, modulations in the beta band (15 to 30 Hz) were generally associated to movement-related functions, such as motor execution, observation, or imagery^26–28^. Since event boundaries are often marked by changes in goal-directed movements and persons with PD were reported to have problems in motion recognition, aberrant beta activity in the PD group might reflect decreased processing of motor cues indicative of event boundaries^27–30^. Besides, beta oscillatory activity was found to be related to content specific maintenance and (re)activation of task relevant information in working memory, so that differences between persons with PD and controls in this frequency band could also point to a disturbance of these functions^31^.

Thus, in the present study we used EEG to assess neuronal activities in different frequency bands related to event boundaries in persons with PD and healthy controls. This was done under the premise that neuronal network activities in various frequency ranges correlate to particular behavioral functions (see above), so that potential group differences on neurophysiological levels could provide insights into processing abnormalities associated with deficient event segmentation in PD.

In the event segmentation task (ES task)^32^, participants watched three movies of around 5–7 min showing a single person performing an everyday activity. While watching the movies, they were asked to indicate by button press when one sub-event had ended and the next one was about to begin. During ES task performance the EEG was recorded and band power changes were analyzed with respect to the individually identified event boundaries.

We hypothesized that persons with PD diverged from healthy controls with respect to the behavioral segmentation performance of the movies. Further, we expected reduced neural activities in the theta and/or alpha band in relation to the set event boundaries due to aberrant event segmentation processing in persons with PD compared to controls^33,34^. Since beta activity is mostly found increased in PD, associated with dysfunctional motor processing, but is further related to the cognitive processes mentioned above, we had no clear prediction about a specific effect in response to event boundaries and explored potential changes in either direction, that is, increased or decreased power in this frequency band in PD^31,35^. The results are discussed in the context of functional concepts for synchronized network activities in different EEG frequency ranges.

## Method

### Participants

Twenty-three medicated subjects with PD (8 female, 15 male) and 25 healthy control subjects (11 female, 14 male) participated in the present study. Participants with overt signs of cognitive impairment [assessed by the Parkinson’s Dementia Assessment [PANDA]; ^36^], symptoms of depression (assessed by the Hamilton Rating Scale for Depression [HRSD]), or having neurological diseases other than PD were excluded. One PD and one healthy control subject had to be excluded due to a technical error in the EEG recording, just like two further healthy control subjects due to severe muscle artifacts in the EEG recording and symptoms of depression, respectively. Thus, 22 participants in the PD group and 22 participants in the control group were included in the final analysis (see Table 1). Both groups did not significantly differ with respect to age, education, and general cognitive functioning (see Table 1). The PD group showed higher depression scores than the control group which is a typical finding in this condition^37^. The participants in the PD group had a mean Hoehn and Yahr disease stage of 2.14 (*SD* = 0.65) and 18.86 points (*SD* = 8.83) in the motor scale (part III) of the Unified Parkinson’s disease Rating Scale (UPDRS). They were diagnosed with PD 6.59 years ago on average (*SD* = 3.86) and their mean L-DOPA daily equivalent dose was 696.41 (*SD* = 357.99). All participants gave their informed consent to the participation in this study, approved by the ethics committee of the Charité in accordance with the Declaration of Helsinki (EA4/022/18).

**Table 1:** Group characteristics.

	PD group (*N* = 22)	Control group (*N* = 22)	Statistics
Mean age in years	65.95 (*SD* = 10.93)	67.14 (*SD* = 7.71)	*t*(42) = 0.41, *p* = 0.681
Sex (female/male)	7/15	9/13	χ^2^ = 0.09, df = 1, *p* = 0.754
Academic background (number of participants)			χ^2^ = 5.76, df = 6, *p* = 0.451
Apprenticeship	4	5	
Professional school degree	4	2	
University of applied science degree	5	6	
University degree	9	7	
Others	1	2	
PANDA cognition score	25.32 (*SD* = 2.82)	26.86 (*SD* = 3.06)	*t*(42) = 1.74, *p* = 0.089
HRSD depression score	6.71 (*SD* = 2.82)	3.09 (*SD* = 3.06)	*t*(42) = − 3.08, *p* = 0.004

*PANDA* Parkinson’s dementia assessment; the cut-off score for cognitive impairment was 14 out of maximum 30 points. *HRSD* Hamilton rating scale for depression; the cut-off score for symptoms of depression was 17 out of maximum 51 points.

### Stimulus material

In the ES task, participants watched three movies showing a single person performing an everyday activity: (1) a woman preparing breakfast in the kitchen (329 s), (2) a man planting two window boxes with flowers in the garden (354 s), and (3) a man preparing a dining room table for a party (375 s) [see ^18,38^]. In addition, one practice movie was presented (i.e. a woman ironing a shirt in the living room, 298 s). The movies were shot without cuts at a fixed camera angle and were presented without sound. Participants were asked to freely segment the movies into meaningful sub-events by button presses, that is, whenever they subjectively judged that one sub-event ended and the next one was about to begin. No instructions regarding the length of the sub-event units were given. Additionally, both groups performed a choice reaction time task (CRT) in which they pressed either the left or right button in response to a point appearing on the left or right side of the screen. Twenty trials were presented, ten for the left and ten for the right side. The CRT task was used as an estimate for the motor influence which the button presses in the ES task had on the neurophysiological data (see Data analysis section for details). Both tasks were presented with the Presentation Software (Version 14.9, www.neurobs.com).

### Procedure

The motor status of the participants with PD was assessed by specialized clinicians during visits in the outpatient clinic for movement disorders at the Charité, Campus Benjamin Franklin, up to 2 weeks before the study day. On the day of the study, participants of both groups gave their informed consent, filled out a demographic questionnaire, and completed the cognitive assessment (i.e. PANDA). They were then prepared for the EEG recording in a separate room. They were seated in front of a 15’’ monitor screen at a distance of 60–75 cm. After recording a resting state EEG of approximately 3 min, both groups completed the CRT task (approximately 2–3 min) and thereafter the ES task (approximately 25 min), while an EEG was recorded simultaneously. When participants had read the standardized instructions of the ES task, they segmented the practice movie (e.g. ironing a shirt). The instructor verbally repeated the task instructions during the practice movie, thereby ensuring correct task understanding. The three movies were then presented in randomized order, with the possibility of breaks between movies. The total study duration was approximately 1.5 h.

### Data acquisition and preprocessing

EEG was recorded with the NeuroScan AQUIRE Application Software (Version 4.5.1, Compumedics USA, Inc.) from 42 Ag/AgCL electrodes positioned in an electrode cap (Easy Cap GmbH, Germany) according to the 10–20 electrode system^39^: FP1, FP2, AF3, AF4, FZ, F3, F4, F7, F8, FC1, FC2, FC5, FC6, FT7, FT8, CZ, C3, C4, T7, T8, CP1, CP2, CP5, CP6, TP7, TP8, PZ, P3, P4, P7, P8, PO3, PO4, PO7, PO8, PO9, PO10, OZ, O1, O2, M1, and M2. The electrooculogram (EOG) was recorded from two electrodes (V + , V–) placed above and below the right eye to measure vertical eye movements. The sampling rate was 500 Hz, the reference electrode was positioned at AFz and the ground at FPz. Impedances were kept below 10 kΩ.

EEG data were preprocessed with the EEGLAB toolbox (v2019.1, Delorme, & Makeig, 2004) in MATLAB (R2020a, The MathWorks Inc., 2014). We re-referenced the electrode signals offline to the mean of the two mastoid electrodes (M1, M2) and corrected for slow drifts and muscle artifacts by applying a bandpass filter between 0.5 and 49 Hz. We extracted epochs time-locked to the button presses of the participants ranging from 1000 ms pre-button press to 1500 ms post-button press in the CRT and ES task, respectively (in the following defined as trials). The continuous resting state data were also epoched into 2500 ms time windows (in the following defined as trials). Trials containing muscle or movement artifacts were rejected based on visual inspection (ES task trial exclusion of 9.98% in the PD group and 12.97% in the control group). Eye movements and blinks were corrected using independent component analysis (ICA) based on the infomax algorithm^40^.

### Data analysis

#### Behavioral analysis

For the CRT task, we compared reaction times (RT) with an independent *t*-test between groups.

For the ES task, the number of identified event boundaries were compared between groups with a mixed model design analysis of variance (ANOVA) including the within-subject factor *movie* (breakfast, garden, party) and the between-subject factor *group* (PD group, control group). Further, we aimed at analyzing if the PD group showed a deviant behavioral segmentation pattern, that is, if they identified event boundaries at different time points in the event sequence compared to the control group. We therefore applied the previously used analysis procedure by Wyrobnik, van der Meer^4^. Each movie was divided into 1-s bins and whenever a participant identified an event boundary in a respective bin (i.e. pressed the button), the value 1 was assigned to this bin, otherwise the value 0 was assigned. This resulted in an individual segmentation pattern of both groups which we defined as the dependent variable in a generalized linear mixed model (GLMM) with a binomial link function. Next, the average number of healthy controls, who identified an event boundary in each bin, was calculated. We defined this resulting segmentation pattern of the control group as normative and used this variable (i.e. *proportion* variable; continuous variable; *z*-transformed for analysis) as a predictor in the GLMM. Note, that for predicting the individual segmentation pattern of the control group, we used an adjusted proportion variable which omitted the respective participant’s segmentation behavior (i.e. *N*-1) to avoid predicting one’s own segmentation behavior. For predicting the individual segmentation pattern of the PD group, we used the proportion variable which included the average segmentation pattern over all healthy controls. In addition, the predictor variables *movie* (breakfast, garden, party) and *group* (PD group, control group) (both contrasts coded) were included in the GLMM and main effects as well as interactions between all predictor variables were analyzed. By-subject intercept and slopes for the variable proportion, movie, and their interaction were included. In case of convergence problems, we removed the correlation parameters and simplified the random effect structure^41^. Nested models were applied to resolve interaction effects involving the effect of group. GLMM analysis was carried out in R-Studio (version 1.2.5033, R Core Team, 2014) with the lme4 and lmerTest packages^42^.

#### Time frequency analysis

Time frequency analyses were conducted in the Fieldtrip toolbox in MATLAB for each movie separately. Power spectra were estimated from the preprocessed EEG single trial data in a time window ranging from 400 ms pre-button press to 1000 ms post-button press in 10 ms steps between 4 and 30 Hz in 1 Hz steps by applying Morlet wavelets with a resolution of seven cycles and the Hanning taper function. The resulting power spectrum was then averaged over trials for each movie as well as across all movies for each subject.

The same procedure was applied to estimate the power spectra associated to CRT task performance, where the data were averaged for left- and right-hand responses per subject. Then, epochs of the ES task were baseline-corrected by subtracting the mean band power of the CRT task with comparably low cognitive demands and a higher degree of basic motor processing than entailed in the ES task. This procedure aimed at reducing the motor influence of the button presses in the ES task leading to results approximately corrected for motor processing. Because all participants reported to be right-handed and used the right hand to indicate event boundaries in the ES task, only the responses of the right hand of the CRT task were used for baseline correction.

Finally, the power spectra of the resting state EEG period were estimated with the same procedure as described above and averaged as well. Then, the mean band power of the resting state EEG period was used for baseline correction to optimally control the data by subtracting the calculated band power of the resting state data form that of the ES task data.

For statistical comparison, spectral power in the theta (4–7 Hz), alpha (8–12 Hz), and beta (15–30 Hz) frequency bands were each compared between the PD and control group with non-parametric cluster-based permutation independent-samples *t* tests^43^ for each movie as well as for the average over all movies. The cluster-based statistics identified significant time–frequency-electrode clusters as the sum of *t* values exceeding a threshold (*p* < 0.05, two-tailed, 1000 random permutations, ≥ 2 channels minimum cluster size).

#### Correlational analysis

We conducted correlational analyses to explore if the behavioral segmentation performance was associated with an aberrant neurophysiological pattern in persons with PD. For this, we first calculated the so-called segmentation agreement score by correlating one’s own individual segmentation behavior to the normative segmentation pattern, that is, to the proportion variable^18^. This score served as an indicator of how normative the individual segmentation pattern was. Given that we did not have one exclusive prediction about how segmentation processing in PD was altered at the neurophysiological level, we extracted the mean power of each frequency band, in which significant differences between groups were found, as an index of the neurophysiological change of segmentation processing in persons with PD. We then computed Spearman correlations between the segmentation agreement score and the mean power of the given frequency bands within each group.

To control whether clinical parameters were associated with the event segmentation pattern in the PD group, we additionally computed Spearman correlations of clinical parameters (i.e. UPDRS motor score, Hoehn and Yahr symptom severity score, dopamine medication dose, HRSD depressions score) with the segmentation agreement scores and mean power in frequency bands with significant group differences, respectively.

## Results

### Behavioral results

The PD group responded significantly slower than the control group in the CRT task (679.14 ms, *SD* = 103.85 versus 558.49 ms, *SD* = 98.89; *t*(42) = − 3.95, *p* ≤ 0.001).

**Figure 1:**
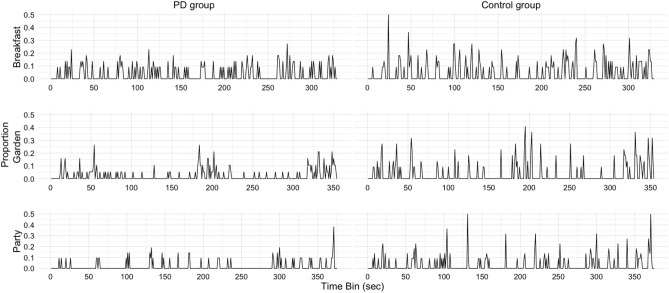
Proportion of participants per group identifying event boundaries in each movie. Proportion values under 0.05 were excluded from the figure for a better display of the results.

With respect to the ES task, we excluded two PD and one control participant in the movie “working in the garden” and one control participant in the movie “preparing breakfast” and “preparing a party table”, respectively, because either the number of identified event boundaries exceeded group and movie means by more than two standard deviations or no event boundary was identified. The analysis of the number of event boundaries indicated a main effect of movie (*F*(2, 76) = 13.31, *p* ≤ 0.001, η_p_^2^ = 0.26) due to more identified event boundaries in the movie “preparing breakfast” than in the movie “working in the garden” (*p* = 0.022). The main effect of group (*F*(1, 38) = 2.67, *p* = 0.111, η_p_^2^ = 0.07) and the interaction between movie and group (*F*(2, 76) = 0.08, *p* = 0.927, η_p_^2^ = 0.002) were not significant. Thus, both groups did not statistically differ with respect to the number of identified event boundaries (PD: 12.59, *SD* = 9.52 versus controls: 18.23, *SD* = 12.55) (see Fig. 1).

To reach convergence in the GLMM analysis, we simplified the random effect structure and removed the correlation and interaction term between the factors proportion and movie (see Table 2). Results of the GLMM indicated significant main effects of proportion and movie and significant interactions between proportion and group, as well as between proportion and movie (see Table 2). To resolve the significant interaction involving the factor group, we conducted a nested model, in which proportion was nested within the effect of group. The nested model showed that the variable proportion (i.e. the normative segmentation pattern derived from the control group) predicted the segmentation behavior of the PD group worse compared to the control group (*b* = 0.21, *SE* = 0.07, *z* = 3.06, *p* = 0.002).

**Table 2:** GLMM analysis results.

Formula: segmentation ~ 1 + proportion * group * movie + (1 + proportion + movie || subject)				
Term	b	SE	z	p
Intercept	− 3.56	0.13	− 26.95	< 0.001
Proportion	0.51	0.03	14.99	< 0.001
Group	0.23	0.26	0.89	0.373
Movie: BF-GA	0.67	0.10	6.51	< 0.001
Movie: PA-BF	− 0.51	0.09	− 5.62	< 0.001
Proportion * group	0.22	0.07	3.23	0.001
Proportion * movie: BF-GA	− 0.23	0.05	− 5.29	< 0.001
Proportion * movie: PA-BF	0.14	0.04	3.42	< 0.001
Group * movie: BF-GA	− 0.18	0.19	− 0.95	0.343
Group * movie: PA-BF	0.12	0.17	0.73	0.464
Proportion * movie: BF-GA * Group	0.07	0.09	0.82	0.415
Proportion * movie: PA-BF * Group	− 0.09	0.08	− 1.07	0.286

Variance components (SD)		Goodness of fit		
Intercept	0.34 (0.59)	Log Likelihood: -7160.3		
Proportion	0.04 (0.19)	REML deviance: 14320.7		
Movie: GA	0.47 (0.68)			
Movie: BF	0.23 (0.48)			
Movie: PA	0.59 (0.77)			

The dependent variable “segmentation” (see Formula) refers to the individual segmentation pattern of each subject. *BF* “preparing breakfast”, *GA* “working in the garden”, *PA* “preparing a party”.

Thus, in line with our assumption persons with PD showed divergent segmentation performance compared to healthy controls.

### Time frequency results

**Figure 2:**
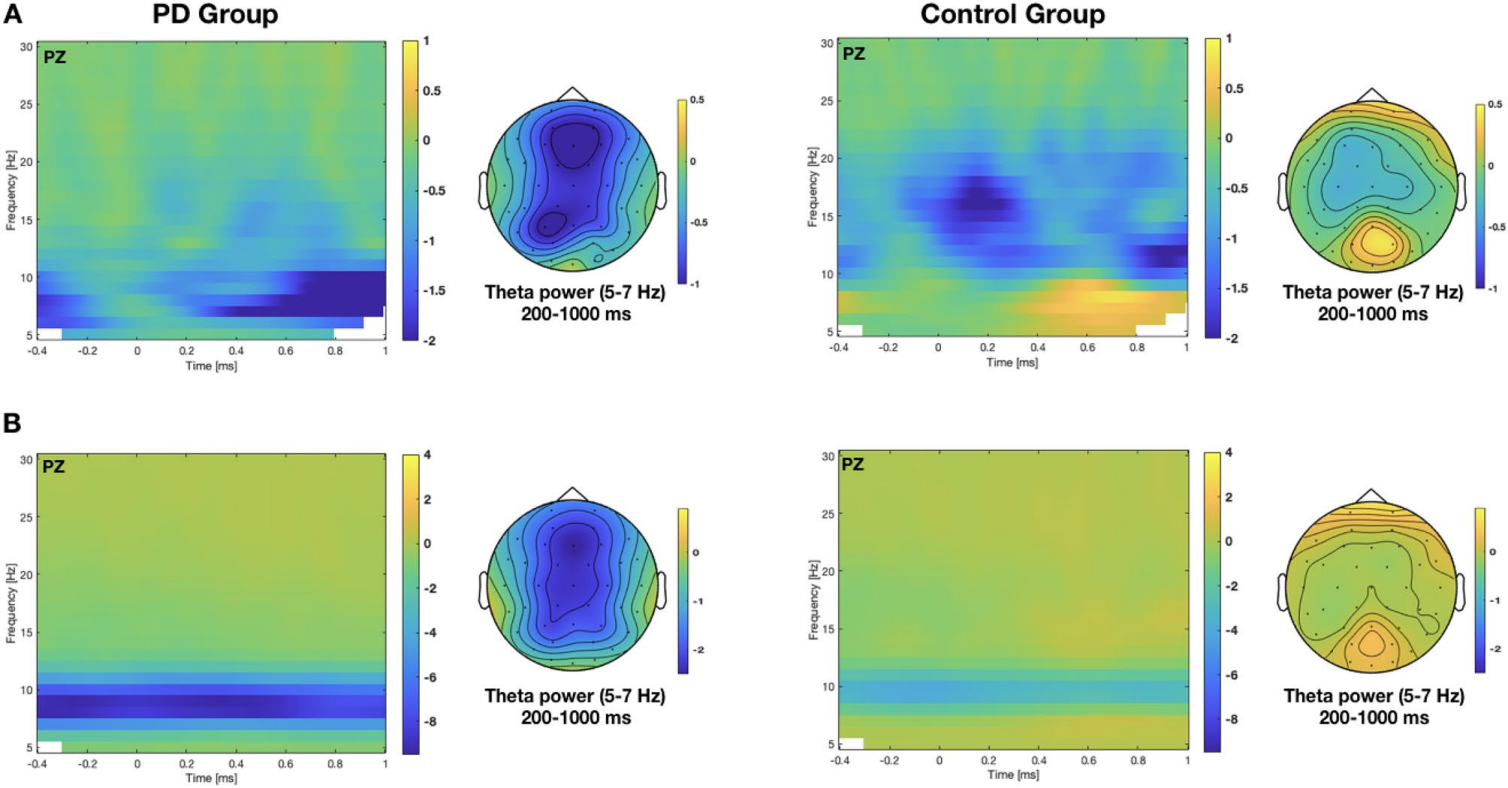
Time frequency results for the average across all movies of the PD and control group. Time frequency representations of the absolute power change (**A**) of the ES task referenced to the CRT task and (**B**) of the ES task referenced to the resting state EEG data at electrode PZ. Zero ms represents the button presses in the ES task, indicating event boundary identification, and button presses in the CRT, but not resting state data. Topographies show the power in the theta (5–7 Hz) band averaged between 200 to 1000 ms post-button press (i.e. frequency range and time period of the significant cluster). The left panel shows the data from the PD group and the right panel those of the control group.

In the theta band (4–7 Hz), the cluster-based permutation tests revealed a significant cluster (*p* = 0.018) over all movies, indicating reduced theta band power in the PD compared to the control group. The cluster in the observed data reflected a significant group difference in the 5–7 Hz frequency band between 200 and 990 ms post-button press over a broad electrode range, including frontal, central, and posterior regions (see Fig. 2). Significant clusters in the 5–7 Hz theta band were also found with respect to the data from the single movies. For instance, in the movie “preparing breakfast” one cluster (*p* = 0.037) extended from approximately − 400 to 0 ms pre-button press (i.e. *before* event boundary identification) and a second cluster (*p* = 0.018) extended from approximately 300 to 990 ms post-button press (i.e. *after* event boundary identification) over a broad electrode range, respectively. In the movie “preparing a party”, one cluster (*p* = 0.019) extended from approximately 260 to 990 ms post-button press over a broad electrode range.

For the alpha power band (8–12 Hz), no cluster reached significance in neither movie, indicating no differences between the PD and control group in the alpha frequency power range.

For the beta power band (15–30 Hz), no cluster reached significance when considering the average over all movies. However, one significant cluster (*p* = 0.04) indicated increased beta band power in the PD compared to the control group in the movie “preparing a party”. The cluster extended from approximately 560 to 1000 ms post-button press over a broad electrode range (e.g. frontal, central, and posterior regions).

When using the band power of a resting state EEG period as a baseline for the ES task, the overall result pattern remained unchanged. That is, a significant cluster in the theta band (4–7 Hz) (*p* ≤ 0.02), but not in the alpha (8–12 Hz) or beta (15–30 Hz) band, was found, indicating reduced theta band power in the PD group compared to the control group (Note, that the resting state data did not differ between groups in the theta (4–7 Hz), alpha (8–12 Hz), or beta (15–30 Hz) bands, indicating similar idling state EEG properties between the groups. Further, due to much higher theta to alpha activity in the resting state compared to the ES EEG, visual depiction of the theta-related group difference in the ES task is difficult, if using the resting state data as baseline, because the absolute change of theta power is relatively small compared to the constantly high starting level in this frequency band.) (see Fig. 2).

In sum, persons with PD showed altered neural processing, that is, reduced theta band power around identified event boundaries compared to healthy controls.

### Correlational analyses results

To test potential associations between the segmentation behavior and the neurophysiological results, we computed Spearman correlations between the segmentation agreement scores and the mean power of each frequency band in which significant differences between groups were found. For the theta band, mean power was extracted between 300 to 900 ms at electrode PZ for the average across movies, in the movie “preparing breakfast”, and in the movie “preparing a party”, and further, mean power was extracted between − 400 to 0 ms in the movie “preparing breakfast”. For the beta band, mean power between 560 to 1000 ms in the movie “preparing a party” was extracted at electrode PZ. Results showed no significant association between the segmentation agreement score and mean power in the theta and beta bands, respectively, for neither group nor movie.

Further, no association between clinical parameters and the segmentation agreement scores / the mean theta band power between 300 and 900 ms at electrode PZ was identified, that is, the segmentation pattern in the PD group was not found to be related to the assessed motor condition, symptom severity, depressiveness, and dopaminergic medication (all *p* > 0.199).

## Discussion

In the present study we aimed at comparing a PD and a healthy control group regarding their event segmentation performance and associated changes of brain activities, expressed as EEG power in different frequency bands. In the event segmentation task, both groups were asked to segment three movies showing a single person performing everyday activities into discrete sub-events. Simultaneously, the EEG was recorded and the given power in the theta, alpha, and beta frequency bands was compared between groups around identified event boundaries. Behaviorally, persons with PD had a segmentation pattern which deviated from that assessed in healthy controls. Further, the PD group showed decreased theta activity (4–7 Hz) in response to event boundaries. No differences between groups were found in the alpha (8–12 Hz) band. Increased beta activity (15–30 Hz) in the PD compared to the control group was found in one of the presented movies, but not in the average across all movies. These findings shall be discussed in the following.

Persons with PD are assumed to show altered event segmentation performance mainly due to deficient basal ganglia (BG) networks, involved in the sequencing of information and updating processes^11,33^. Indeed, the present results indicate that persons with PD deviate from a control group in identifying typical (i.e. normative) points of event boundaries in everyday activities, thus supporting the assumption of PD-related impairment of event segmentation^4^. Interestingly, in previous studies theta activity was associated with the maintenance and manipulation of working memory information in temporal sequencing processes and, further, with processing unexpected events and prediction errors^23,44–47^. Accordingly, poor sequential working memory performance in PD was linked to decreased alpha and theta power^34^. With respect to the event segmentation theory, decreased theta activity in the current task could indicate difficulties to build stable event models in working memory, when bottom-up perceptual input has to be aligned with stored event representations in persons with PD. In this sense, the finding would point to dysfunctions of generating accurate predictions about pending or incipient occurrences, resulting in decreased updating processing of the unexpected events. Besides, in the literature neural activity in the theta frequency range was also associated with the retrieval and storage of episodic long-term memory contents, so that reduced theta marking an event boundary in PD could indicate impaired retrieval of event information from long-term memory to be matched with the perceived input or, alternatively, difficulties to store recently perceived event information in long-term memory^8,21,23,45^. In this context, it is of note that dysfunctional event segmentation in PD was shown to be associated with declined recall of the presented sub-event order^4^.

Two minor findings refer to results which were only present in a subset of stimuli, that is, in one movie, but not in the average across all movies. Firstly, reduced theta power activity started before participants indicated event boundaries (i.e. − 400 to 0 ms) in the PD group compared to controls in the preparing-breakfast-movie. Whether this reflects altered event boundary processing or other operations, is not entirely clear. Noteworthy, event boundary perception slightly preceded button presses, given that a reaction time has to elapse until the response. Thus, theoretically the theta-related results could reflect altered pre-processing of the motor response in PD. Yet, theta is mostly viewed as a correlate of cognitive processing, for example, of working memory and episodic encoding and retrieval functions, rather than of preparatory motor operations^22,23^. Therefore, we consider a relation of the result to impaired boundary recognition as likely. Secondly, beta activity was higher in the PD compared to the control group in one out of three movies after button presses. PD-related excess of neuronal oscillations in this frequency range has mostly been related to impaired motor processing^48,49^. In the present study, however, enhanced beta activity was found despite the attempt to minimize the influence of a PD-related "motor signature" by subtracting the signal of the choice reaction time task from that in the ES task (implying identical button presses for the indication of event boundaries). One explanation for the residual beta enhancement could be that oscillatory activity in this frequency range does not only relate to the performance of movement, but also to its perception^28,50^ and that PD impacts on both motor execution and recognition^29,51^. Accordingly, the current results could indicate that persons with PD differed from controls in the processing movement-related sensory cues typically denoting event boundaries^30,52^. Alternatively, with respect to further functional roles attributed to beta activity, the obtained power increase in PD could reflect altered working memory processing, in this case related to the maintenance of a stable event model^31,53^. For example, enhanced beta synchronization could indicate compensatory mechanisms for the primary deficit of establishing, keeping, or updating event models, presumably related to the identified theta reduction and, thus, poor event segmentation.

### Limitations

In this study, persons with PD were examined under their regular medication, which constrains inferences about potential dopaminergic mechanisms of event segmentation. To overcome this shortcoming, future studies could focus on additional comparisons with data assessed after drug withdrawal, which, however, puts additional strain on persons with PD. Further, the exact functional impact of reduced theta activity in PD remains vague. Of note, neural oscillations in this frequency range in anterior cingulate cortex (ACC) and the medial prefrontal cortex (mPFC) were associated with the maintenance of serial information in working memory and the processing of prediction errors^44,46^, whereas in hippocampal areas they were rather related to the retrieval and storage of (event) memory content^22^. In this regard, source localization based on recordings with a denser electrode array could provide better insight into the regional distribution and the particular dysfunction related to reduced theta expression in PD. Another issue refers to the identification of event boundaries by button presses. As movement deficits in PD go along with neurophysiological changes^54^, motor influence on the EEG data in the ES task cannot be ruled out. We aimed at reducing this potential impact by subtracting the corresponding signal from a CRT task with comparably low cognitive and proportionally higher motor load. However, this might have included neurophysiological correlates of non-motor operations only present in the CRT. In this regard, it is worthwhile to note that significantly reduced theta power in the PD group in response to event boundaries was also seen when a resting EEG period was used as a baseline. Methodologically, data driven approaches^8,55^ may allow to analyze brain activity during event segmentation without active button presses, and further studies could use these approaches in persons with PD. Having said that, these methods mainly rely on algorithms, which identify interindividual similarities of temporal and spatial signal patterns, which appears problematic in the current context, since PD genuinely alters the neurophysiological signature of event processing. Finally, some findings in the current study were found in a subset of stimuli only (i.e. in one movie). These results may be followed up in future experiments, for example directly addressing PD-related difficulties of event boundary detection and potential associations with beta activity changes as a function of movement perception.

## Conclusions

The main finding of abnormal event segmentation associated with diminished theta activity in persons with PD compared to healthy controls supports the idea of a matching deficit of ongoing sensory input with stored event knowledge and impaired updating processes of event models in working memory. This interactional deficit between memory-related and perceptual functions could result in imprecise prediction of environmental scenarios, a potential source of multifold problems which persons with PD can develop in daily routines.

## Data Availability

The data set used and analyzed during the current study is available from the corresponding author on reasonable request.
